# Pathophysiology of juvenile idiopathic arthritis induced pes planovalgus in static and walking condition—A functional view using 3d gait analysis

**DOI:** 10.1186/s12969-015-0022-z

**Published:** 2015-06-10

**Authors:** Josephine Merker, Matthias Hartmann, Florian Kreuzpointner, Ansgar Schwirtz, Johannes-Peter Haas

**Affiliations:** Department of Biomechanics in Sports, Faculty of Sports and Health Sciences, Technische Universität München, Georg-Brauchle-Ring 60/62, 80992 Munich, Germany; German Center for Pediatric and Adolescent Rheumatology, Gehfeldstrasse 24, 82467 Garmisch-Partenkirchen, Germany

**Keywords:** Juvenile idiopathic arthritis, Pes planovalgus, Gait analysis, Foot kinematics, Oxford Foot Model

## Abstract

**Background:**

Patients suffering from juvenile idiopathic arthritis (JIA) frequently have affected ankle joints, which can lead to foot deformities such as pes planovalgus (JIA-PPV). Usually, JIA-PPV is diagnosed by examining the foot in non-weightbearing or in weightbearing, static condition. However, functional limitations typically appear during dynamic use in daily activities such as walking. The aim of this study was to quantify the pathophysiology of JIA-PPV in both static and dynamic condition, i.e. in upright standing and during the stance phase of walking using three-dimensional (3d) gait analysis.

**Methods:**

Eleven JIA patients (age = 12y) with at least one affected ankle joint and fixed pes planovalgus (≥5°) were compared to healthy controls (CG) (n = 14, age = 11y). Kinematic and kinetic data were obtained in barefoot standing and walking condition (1.1–1.3 m/s) with an 8-camera 3d motion analysis system including two force-plates and one pressure distribution plate. All participants were prepared using reflecting markers according to the Oxford Foot and Plug-in-Gait Model. Results were compared using the Mann–Whitney-*U*-test and Wilcoxon signed-rank test (*p* < 0.05).

**Results:**

In comparison to CG, JIA-PPV had an excessive hindfoot/tibia eversion (*p* < 0.001) and a forefoot/hindfoot supination (*p* < 0.001) in both static and walking condition. JIA-PPV showed a greater hindfoot/tibia eversion during walking (midstance) compared to standing (*p* = 0.021) in contrast to CG. The arch index, measured by plantar pressure distribution, indicates a reduced arch height in JIA-PPV (*p* = 0.007). Patients had a lower maximum dorsiflexion of hindfoot/tibia (*p* = 0.001) and a lower plantarflexion of forefoot/hindfoot (*p* = 0.028), both when standing and walking. The kinetic results showed lower maximum ankle dorsiflexion moments (*p* < 0.037) as well as generated ankle power (*p* = 0.086) in JIA-PPV.

**Conclusions:**

The pathophysiology of JIA-PPV during walking indicated that excessive hindfoot eversion produces accessory symptoms such as a reduced arch height, increased forefoot supination and reduced propulsion effect of the ankle. Muscular and coordinative insufficiency caused by arthritis can lead to the observed increased hindfoot eversion from static to dynamic condition. Conventional static or passive foot examination techniques probably underestimate deformity in JIA pes planovalgus. 3d gait analysis might be helpful in early diagnosis of this condition, especially in JIA patients with affected ankle joints.

## Background

**Fig. 1:**
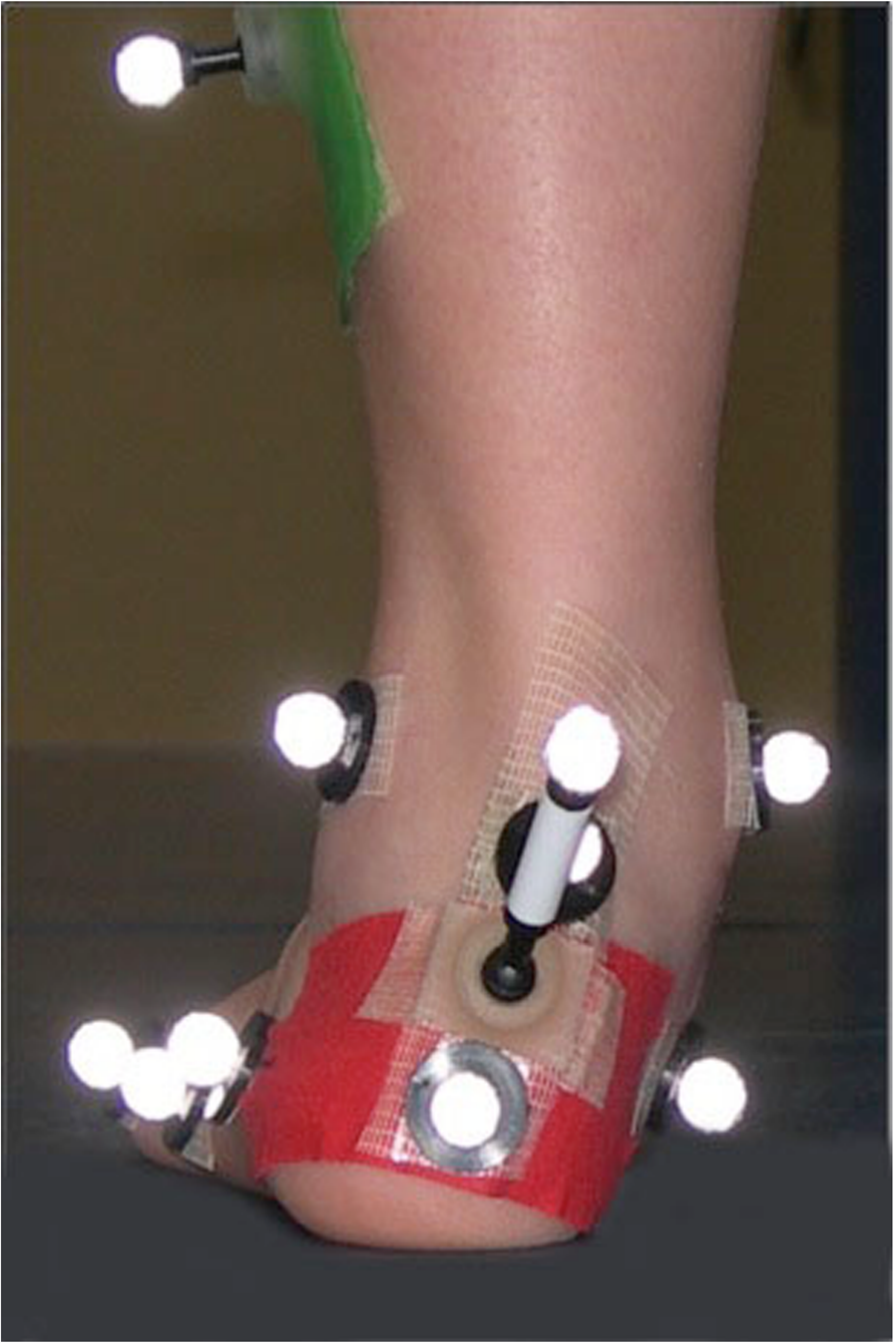
Juvenile idiopathic arthritis induced pes planovalgus in standing position. The foot is prepared with skin markers according to the Oxford Foot Model

Juvenile idiopathic arthritis (JIA) is the most frequent chronic rheumatic disease in childhood [1]. It causes a chronic inflammation of the joints, with increased production of synovial fluid, painful swellings and reflexive pain-relieving positions [2]. These often result in a muscular imbalance, malposition and eventually in fixed deformities such as pes planovalgus (PPV) (Fig. 1) [3]. In general, pes planovalgus is a frequently observed foot malposition in children and adolescents which may develop due to various reasons. Especially in JIA, the incidence is high if inflammation includes the talonavicular and subtalar joints [4].

There are several examination methods for diagnosing JIA-PPV. Usually, the foot and its mobility are examined in an observational setting during clinical routine examinations in a non-weightbearing or static, weightbearing condition. Therefore, conventional methods such as the neutral-zero method and static radiographs are used. Furthermore, laboratory examinations and imaging methods, for example ultrasonography, radiographs or magnetic resonance imaging, detect inflammatory activity of joints.

However, the functional limitations of JIA-PPV are not always readily observable in static examinations, and functional limitations typically appear during dynamic use when walking. Special foot models, such as the Oxford Foot Model (OFM) [5], allow detailed and valid examination of foot deformities during upright standing and walking [6–13]. The relative motion between tibia, hindfoot, forefoot and hallux segments determines the kinematics of the foot. The use of 3d motion analysis enables a quantitative evaluation of the foot, which allows further analysis compared to qualitative observations in the routine clinical setting. An early functional diagnosis is important to prevent serious foot deformities with possible negative consequences for neighboring joints of the lower extremity and the complete posture.

The aim of this study is to quantify the pathophysiology of JIA-PPV during static condition and during the stance phase of walking using 3d gait analysis for the first time. It is hypothesized that JIA-PPV is associated with an excessive hindfoot eversion, a lower longitudinal arch, a forefoot supination and a reduced propulsion effect of the ankle. In addition, differences are expected between results of static and dynamic condition in JIA-PPV compared to an age-matched group of healthy controls.

## Methods

### Participants

All JIA patients recruited were inpatients from the German Center for Pediatric and Adolescent Rheumatology in Garmisch-Partenkirchen. The patient group included six girls and five boys aged eight to sixteen years (Table 1) with at least one affected ankle joint and fixed PPV. Further joints of the lower extremities affected by JIA were documented but disregarded for the following analysis (Table 2). Assessment of the foot deformity was performed independently by an experienced physician and a physiotherapist. Their examinations were the basis for the decision as to which PPV was suspected to result from JIA. In addition, the inclusion criteria for patients were:

- Previously documented ankle joint arthritis (ultrasound imaging was used for identifying joint effusion and laboratory examinations to detect inflammation),
- hindfoot eversion of more than 5° during standing,
- heel valgus position while on tiptoe (a criterion for fixed PPV),
- no previous surgery on the lower extremities,
- no intra-articular injection into affected joints within the preceding four weeks,
- ability to walk freely (no crutches).

**Table 1:** Anthropometrical characteristics and spatio-temporal parameters of JIA pes planovalgus patients (JIA-PPV) and control group (CG)

	JIA-PPV		CG		
	(n = 11)		(n = 14)		
Parameter	Median	Q25/Q75	Median	Q25/Q75	*p*-value
Age (y)	11.7	9.7/14.0	10.9	9.9/12.6	0.784
Height (m)	1.46	1.37/1.59	1.47	1.4/1.6	>0.999
Weight (kg)	44.1	31.0/55.8	39.9	30.4/45.4	0.511
Body Mass Index (kg/m^2^)	19.0	15.9/21.8	17.7	15.4/19.1	0.291
Walking speed (m/s)	1.14	1.09/1.21	1.28	1.20/1.34	0.075
Step length (m)	0.60	0.51/0.66	0.62	0.59/0.66	0.291
Step width (m)	0.10	0.08/0.11	0.08	0.06/0.10	0.166
Foot off (%)	59.4	59.0/60.2	59.3	58.7/59.9	0.477

^*^Statistically significant as *p* < 0.05

**Table 2:** Characteristics of JIA pes planovalgus patients (JIA-PPV)

		JIA-PPV		
Parameter		n	Median	Q25/Q75
JIA sub-types				
	Systemic arthritis	2	-	-
	Persistent oligoarthritis	2	-	-
	Extended oligoarthritis	4	-	-
	Polyarthritis (RF neg.)	2	-	-
	Enthesitis-related arthritis	1	-	-
JIA affected joints of the lower extremities				
	Hip left/right	5/4	-	-
	Knee left/right	9/9	-	-
	Ankle left/right	10/10	-	-
	Midfoot left/right	3/3	-	-
	Toe left/right	3/2	-	-
Drugs				
	Non-steroidal anti-inflammatory drugs	4	-	-
	Disease-modifying anti-rheumatic drugs	9	-	-
	Biologicals	7	-	-
Physician global assessment of overall disease activity (VAS 0-10 cm)		11	4.1	0.9/7.1
Duration of disease (y)		11	7.5	5.6/10.7
Pain intensity (VAS 0-10 cm)		11	1.5	0.0/1.6

JIA was diagnosed according to the 2004 ILAR classification criteria [14], and the different JIA sub-types of the patients included in the study are listed in Table 2. The physician global assessment of overall disease activity (variable of ACR Pediatric core set [15]) was 4.1, and the median duration of disease was 7.5 years (Table 2). The patients reported having a pain intensity of 1.5 according to Visual Analog Scale (VAS), and almost all patients take several pain and inflammation relieving drugs (Table 2).

For comparison, a control group (CG) of fourteen voluntary, healthy children (10 girls, 4 boys) in the same age range was examined (Table 1). None had any rheumatic, orthopaedic or neurological diseases nor any lower limb surgeries or orthopaedic insoles. These criteria have been requested in a special questionnaire, which was developed for this study.

The research was conducted in accordance to the Declaration of Helsinki. Ethical approval for the study was granted by the university’s research ethics committee (Reference 351/14).

### Data collection and processing

Kinematic and kinetic data were collected with an eight camera motion capture system (Vicon, Oxford, UK; sampling rate: 200 Hz) and two force-plates (Advanced Mechanical Technology Inc, Watertown, USA; sampling rate: 1000 Hz). Plantar foot pressure data were obtained using one pressure distribution plate (Novel Emed, Munich, Germany; 4 sensor/cm^2^; sampling rate: 100 Hz), which was embedded in the walkway such as the two force-plates.

The analysis of foot movement was performed according to a standardized examination protocol by one experienced examiner. For the analysis, 44 infrared reflective markers (diameter: 9.5 mm) were attached to the lower extremities of the subjects as required by the OFM [5] and Plug-in-Gait Model (PIG) [16]. Although both legs of each participant were measured, data from only one leg were analyzed. In JIA-PPV, the dominant PPV was of interest (left n = 9; right n = 2). The dominance was identified by a physiotherapist determining the maximum heel valgus in standing position. In CG, a randomly assigned foot was evaluated (left: n = 6; right: n = 8).

**Fig. 2:**
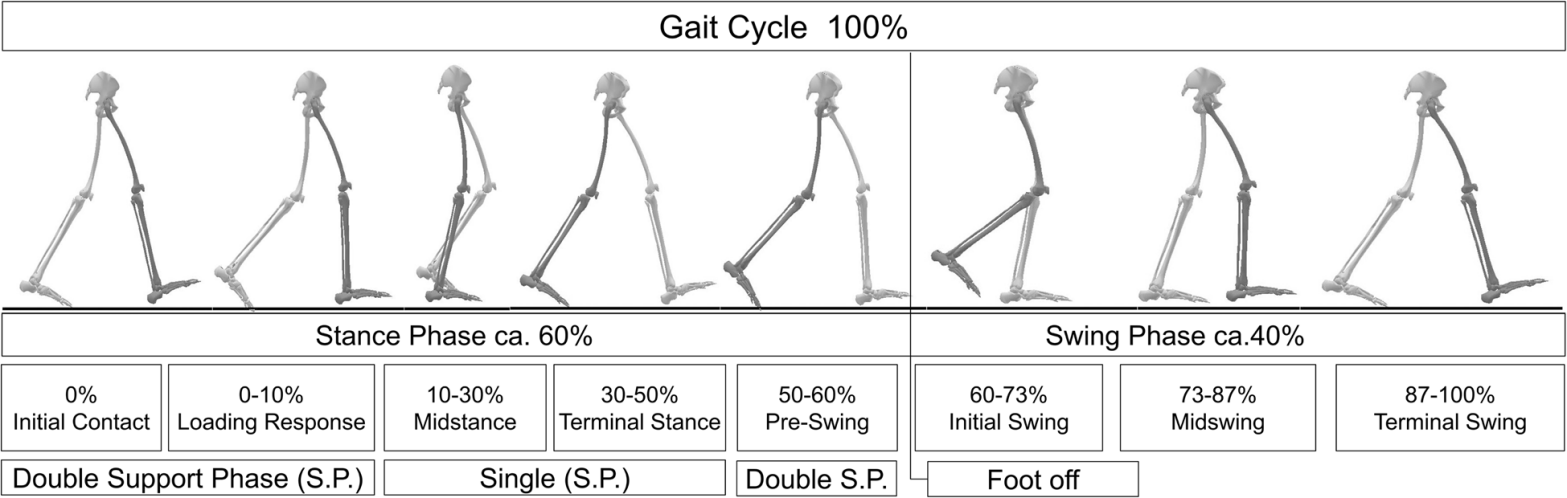
Classification of a normal gait cycle (modified to Perry 1992, p. 2–4) [20]

One static trial was captured before the dynamic assessment of JIA-PPV in walking condition started. For the static trial, the participants were required to stand in a normal, relaxed upright standing in shoulder-width stance. Afterwards, the participants were asked to walk along a nine-meter walkway at their comfortable walking speed. All subjects had to complete at least two attempts in order to get accustomed to the test situation of the dynamic measurement. The starting position of the walk was adjusted by the examiner to allow the participant to hit the force-plates and pressure distribution plate. Ten successful trials with left and right foot contacts on the force-plates were collected. Those were scaled in separated gait cycles and normalized to 100 %. In general, a gait cycle is defined as one stance and one swing phase; starting with the initial contact and ending with the next initial contact of the same leg [17] (Fig. 2). The final dataset includes five out of ten valid gait cycles of each subject. They were randomly selected and visually inspected by the examiner with the demand for each individual to be as normal as possible. In case of an untypical event, the trial was excluded and another one was included. For dynamic pressure measurements, a total of five left and five right foot contacts were recorded and averaged per side.

**Fig. 3:**
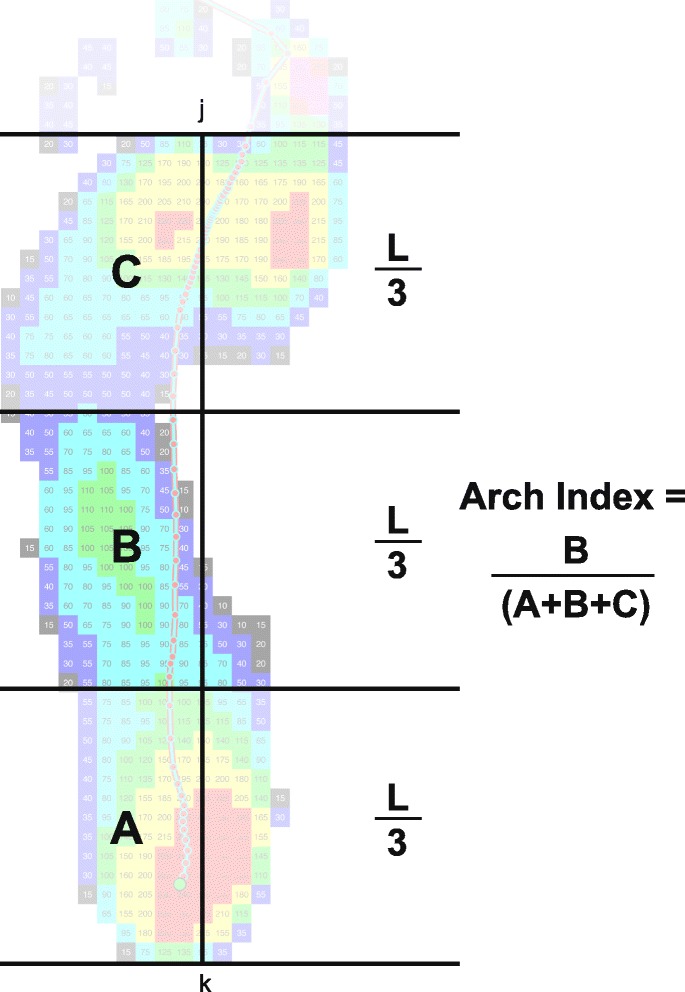
Example of a footprint and the calculation of the Arch Index. The point j and k represent the length of the footprint, excluding the toes. A, B and C represent equal thirds, which are divided by parallel lines perpendicular to the line jk. The arch index is calculated as the ratio of the midfoot area (B) relative to the total area (A + B + C) excluding the toes [19]
- Foot progression angle (PIG parameter)
  - in static condition
  - max in mid stance
- Forefoot to hindfoot motion (FF/HF; OFM parameter)
  - supination/pronation and dorsiflexion/plantarflexion in static condition
  - max supination, max pronation and ROM in stance phase
  - max dorsiflexion in terminal stance, max plantarflexion in pre-swing and ROM in push-off
- Ankle kinetics (PIG parameter)
  - max of ankle joint dorsiflexion moment
  - max generated ankle joint power

To verify the alterations in foot motion in JIA-PPV during upright standing and during stance phase of walking, the following parameters were examined:

- Hindfoot to tibia motion (HF/TB; OFM parameter)
  - inversion/eversion and dorsiflexion/plantarflexion in static condition
  - inversion/eversion at initial contact, maximum (max) eversion, max inversion and range of motion (ROM) in stance phase
  - max dorsiflexion in terminal stance, max plantarflexion in pre-swing and ROM in push-off
- Medial longitudinal arch
  - arch height (AH; OFM parameter) normalized to foot length in static condition
  - minimum (min), max and ROM AH in mid stance
  - arch index (AI; plantar pressure parameter) as ratio of midfoot area relative to total area excluding the toes [19] (Fig. 3)

### Statistical analysis

The non-parametric statistical Mann–Whitney-*U*-test was used to determine differences between JIA-PPV and CG, as not all data were normally distributed. Values of maximum HF/TB eversion, minimum AH and maximum foot progression angle were compared in static and walking condition using the Wilcoxon signed-rank test. For descriptive indices, the median plus quartile 25 and 75 were used. Differences with a *p-value* smaller than 0.05 were accepted as statistically significant. SPSS 22.0 was used for statistics (IBM, Armonk, USA).

## Results

### Group characteristics and spatio-temporal parameters

Table 1 summarizes the group characteristics and results of spatio-temporal parameters. No significant differences in anthropometric characteristics and spatio-temporal parameters between JIA-PPV and CG were found (*p* > 0.05).

### Kinematics of static condition

Results are presented in Table 3. In upright standing, some significant differences were observed between the two groups: In JIA-PPV, the hindfoot is more everted (*p* < 0.001) and less dorsiflexed (*p* = 0.033) in relation to the tibia. Furthermore, the forefoot is more supinated (*p* < 0.001) and dorsiflexed (*p* = 0.014) in patients. The medial longitudinal arch height (*p* = 0.274) and foot progression angle (*p* = 0.511) were similar in both groups.

**Table 3:** Kinematic and kinetic outcome values of upright standing and stance phase of walking. Data of JIA pes planovalgus patients (JIA-PPV) and healthy control group (CG)

			JIA-PPV (n = 11)			CG (n = 14)		
Parameter			Median	Q25/Q75	*p*-value	Median	Q25/Q75	*p*-value
Hindfoot to tibia motion (°)								
	DF/PF	static	1.8	−2.5/4.1		4.0	2.2/6.9	0.033*
		dorsiflexion (max in TSt)	2.5	−0.1/5.7		10.0	6.2/11.2	<0.001*
		plantarflexion (max in PSw)	−14.1	−17.7/-5.5		−7.1	−11.3/-4.9	0.120
		ROM (TSt to PSw)	15.4	17.7/10.1		17.0	19.4/14.5	0.434
	IV/EV	static	−9.2	−12.5/-5.3		−0.5	−3.4/1.4	<0.001*
		in/eversion (IC)	−6.4	−9.1/-2.3		4.0	0.7/6.6	<0.001*
		eversion (max in MSt)	−11.1	−15.0/-9.0		−2.4	−3.2/-0.8	<0.001*
		inversion (max in PSw)	−0.2	−5.1/2.1		10.5	5.2/12.8	<0.001*
		ROM (IC to MSt)	5.4	7.1/4.3		5.7	7.6/3.7	0.767
		ROM (MSt to PSw)	11.0	8.3/13.6		12.2	9.6/15.0	0.317
		static vs. max in MSt	−9.2	−12.5/-5.3	0.021*	−0.5	−3.4/1.4	0.14
			−11.1	−15.0/-9.0		−2.4	−3.2/-0.8	
Forefoot to hindfoot motion (°)								
	DF/PF	static	10.1	6.1/14.2		5.6	4.1/6.8	0.014*
		dorsiflexion (max in TSt)	18.0	11.5/21.2		13.4	10.7/14.5	0.058
		plantarflexion (max in PSw)	3.2	−4.2/5.9		−4.8	−6.8/-2.7	0.005*
		ROM (TSt to PSw)	14.8	19.7/12.1		17.7	19.0/15.3	0.244
	SP/PR	static	14.3	12.4/19.5		5.5	2.0/8.2	<0.001*
		supination (max in LR)	17.8	12.0/20.9		5.4	1.8/7.4	<0.001*
		pronation (max in TSt)	8.8	7.1/13.9		−0.1	−1.5/1.8	<0.001*
		ROM (LR to TSt)	7.0	8.4/4.5		4.3	6.2/3.6	0.006*
Medial longitudinal arch								
	AH (%)	static	20.7	19.0/22.0		21.6	20.5/22.3	0.274
		minimum (MSt)	21.4	18.0/23.6		20.4	19.5/21.5	0.767
		maximum (MSt)	21.6	18.5/23.8		21.3	20.1/22.2	0.809
		ROM (min to max MSt)	0.5	0.2/0.8		0.6	0.4/0.9	0.501
		static vs. min in MSt	20.7	19.0/22.0	0.929	21.6	20.5/22.3	0.096
			21.4	18.0/23.6		20.4	19.5/21.5	
	AI	(MSt)	0.25	0.23/0.27		0.22	0.17/0.23	0.007*
Foot progression angle (°)								
		static	−5.8	−8.4/-0.9		−6.1	−9.1/-3.7	0.511
		maximum (MSt)	−6.0	−10.5/-3.0		−4.0	−6.8/-1.0	0.267
		static vs. MSt			0.594			0.158
Ankle kinetics								
		Dorsiflexion moment in late stance phase (Nm/kg)	1.3	1.1/1.4		1.4	1.3/1.5	0.037*
		Power in late stance phase (W/kg)	3.3	2.1/4.3		3.9	3.5/4.4	0.08

*DF/PF*, Dorsiflexion/plantarflexion; *IV/EV*, Inversion/eversion; *SP/PR*, Supination/pronation; *AH*, Arch height OFM; *AI*, Arch index; *ROM*, Range of motion; *max*, maximum; *min*, minimum; *IC*, Initial contact; *LR*, Loading response; *MSt*, Mid stance; *TSt*, Terminal Stance; *PSw*, Pre-swing

*Statistically significant as *p* < 0.05

### Kinematic gait data

**Fig. 4:**
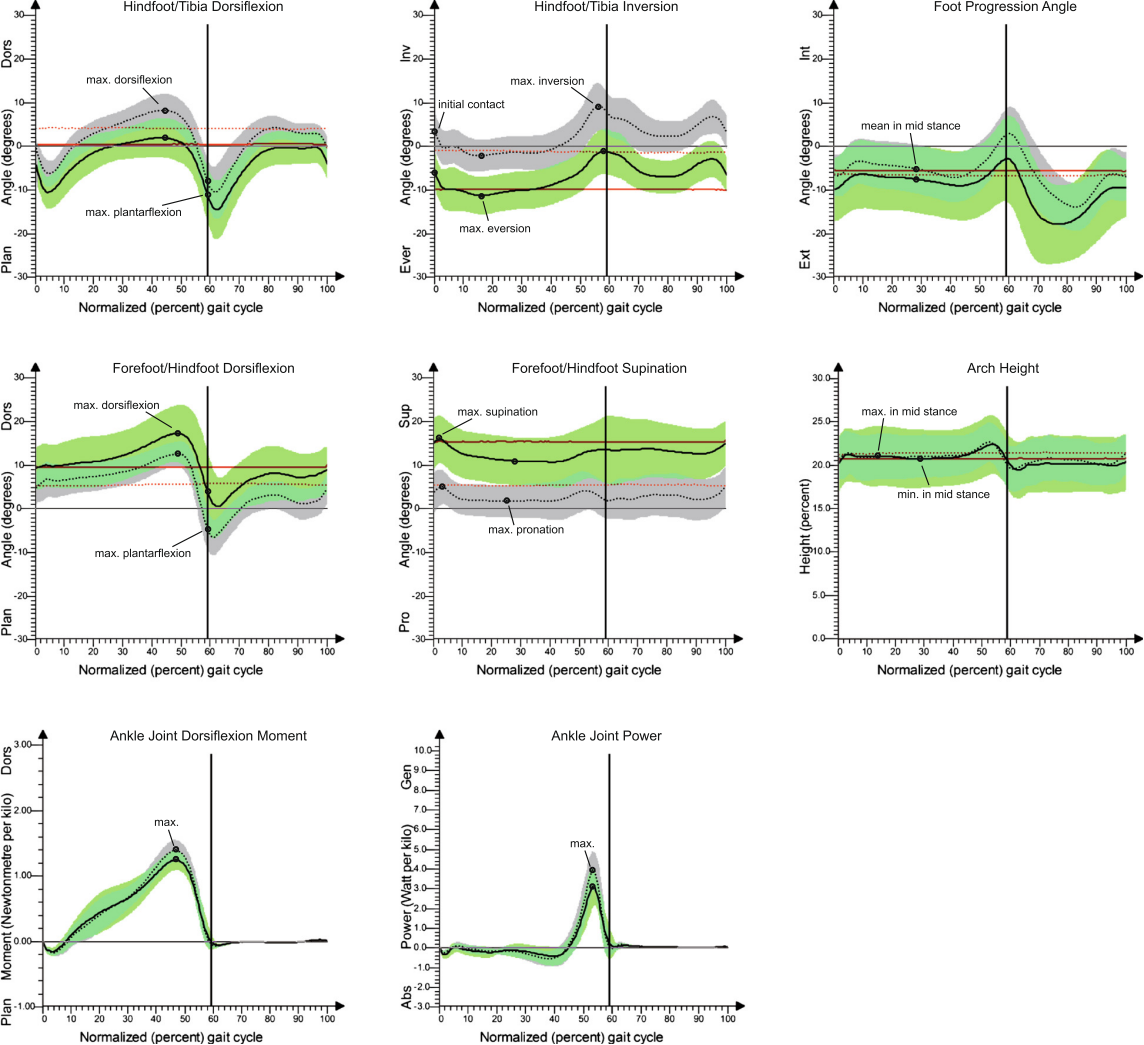
Foot kinematics and kinetics. The solid red line (mean) represents the JIA pes planovalgus patients (JIA-PPV) and the red dotted line (mean) represents the healthy control group (CG) in static condition. In walking condition, the solid line (mean) with green shade (SD) represents JIA-PPV and the dotted line (mean) with gray shade (SD) represents CG. The maxima and the range between them are the points of interest. All angles were time-normalized to the percent of the gait cycle. The vertical lines divide stance and swing phase. Significant differences are indicated by *p*-value < 0.05

Table 3 contains test statistics for all kinematic parameters, and Fig. 4 shows the inter-segment angle profiles of all measurement outcomes. The kinematic data were only evaluated during the stance phase of walking, which was almost identical in duration across both examination groups (JIA-PPV Mdn = 59.4 % vs. CG Mdn 59.3 %, *p* = 0.477).

The comparison of patients with CG revealed significant differences in the frontal hindfoot/tibia motion: At initial contact, the hindfoot was already in 6° eversion in JIA-PPV (*p* < 0.001). In mid stance, the patients reached a maximum hindfoot eversion of 11° compared to 2° eversion of CG (*p* < 0.001). The peak inversion appeared in the late stance phase (pre-swing) and was significantly smaller in JIA-PPV (*p* < 0.001). However, the total eversion and inversion ROM were similar in both groups. JIA-PPV showed a significantly lower peak dorsiflexion of hindfoot to tibia motion (*p* < 0.001) in sagittal plane during terminal stance. There were no differences in peak plantarflexion in pre-swing and in ROM between maximum dorsiflexion and plantarflexion.

The minimum, maximum and ROM of medial longitudinal arch height normalized to foot length were alike in mid stance for patients with JIA-PPV and healthy children. In contrast, the calculated arch index (dimensionless) of foot pressure measurements differed significantly between the groups (*p* = 0.007). The patients had an increased arch index value, which is associated with a lowered medial longitudinal arch [18]. In the foot progression angle, the maximum external rotation of the foot did not differ between JIA-PPV and CG in mid stance.

In frontal plane during loading response, a significant increase of forefoot supination was observed in patients (*p* < 0.001). Large differences were noted in terminal stance as well, where JIA-PPV showed less maximum pronation (*p* < 0.001). The total pronation ROM was significantly increased in JIA-PPV (*p* = 0.006). In sagittal plane, the patients demonstrated a significantly lower peak forefoot to hindfoot plantarflexion motion (*p* = 0.005) during pre-swing. However, peak forefoot dorsiflexion in terminal stance and the range of maximum dorsiflexion to maximum plantarflexion did not show any significant differences comparing both groups.

### Kinematics of static condition versus gait data

The magnitude of maximum hindfoot/tibia eversion was significantly greater for mid stance of walking than for upright standing in JIA-PPV (*p* = 0.021) (Table 3). No differences could be observed in hindfoot/tibia eversion for CG for both conditions (static vs. walking). The data of minimum medial longitudinal arch height and foot progression angle of JIA-PPV and CG were similar during upright standing and mid stance of walking.

### Kinetic data

On average, JIA-PPV showed a significantly lower maximum in the ankle joint dorsiflexion moment (*p* = 0.037) in terminal stance (Table 3, Fig. 4). Analysis of the peak generated ankle joint power at push-off revealed trends for a reduction in patients with respect to controls (*p* = 0.080) (Table 3).

## Discussion

The purpose of this study was to evaluate the pathophysiology of JIA induced pes planovalgus (JIA-PPV) during upright standing and stance phase of walking using 3d gait analysis. First, we found various considerable deviations in kinematics and kinetics of JIA-PPV compared to CG. The patients show the typical characteristics generally described in JIA-PPV [4], with the exception of external foot rotation. Consequently, the hindfoot was more everted and less dorsiflexed. In addition, JIA-PPV had a larger supination and dorsiflexion of the forefoot in both examination conditions. The plantar pressure data demonstrated a lowered medial longitudinal arch of JIA-PPV during walking. The propulsion effect of the ankle is also reduced in patients. Secondly, we found a significantly increased hindfoot eversion from static to dynamic condition in JIA-PPV.

We consider these findings to be independent of the slightly different walking speed: Patients walked about 0.14 m/s slower than CG, but differences were statistically nonsignificant. As there were no differences in the anthropometric characteristics and the results of foot kinematics of CG were most similar to those found by other authors [5, 7, 12, 19–21], we believe that the observed differences between the groups can be referred to a successful determination of PPV characteristics using 3d motion capturing.

### Excessive hindfoot/tibia eversion

As expected, JIA-PPV showed an increased maximum hindfoot to tibia eversion position in upright standing and a considerable shift in eversion of about 9° - 11° during walking compared to CG. The peak hindfoot eversion is greater in walking than in upright standing for JIA-PPV. This shows that JIA-PPV is not solely a static foot deformity, and evaluation of JIA-PPV only in static condition would be insufficient. In upright standing and walking, the peak eversion in JIA-PPV was significantly higher than in CG. An eversion maximum of up to 5° is considered to be physiological, and it is an important part of shock absorption of the body during movement such as walking [22, 23]: The transverse tarsal joints are unlocked, the arch height is lowered and the foot becomes flexible so that shock absorption is possible [24].

Clinically, hindfoot eversion appears to be very prominent in the pes planovalgus population, but recent studies report inconsistent findings [12, 13, 25]. Only a few studies using gait analysis in flat-footed populations also found increased hindfoot peak eversion motion during walking [12, 13]. Furthermore, Hoesl et al. [13] described a significantly decreased peak inversion in late stance, which was also found in this study. However, no differences were found in the frontal hindfoot ROM between both examination groups, which is comparable to the findings of Twomey et al. [25]. The overall shape of the inter-segment foot angle profiles of JIA-PPV can be compared quite well to those of CG, but a closer look reveals that the hindfoot remains in a “fixed eversion position”. The neutral hindfoot position of the physiologically stated value of 5° cannot be reached in JIA-PPV, neither in standing nor in the stance phase of gait.

### Medial longitudinal arch flattening, but no external foot rotation

The distinctive hindfoot eversion position in JIA-PPV could also lead to a significantly lower medial longitudinal arch height and an external rotation of the foot. This was not confirmed during standing and walking by the OFM and PIG data. However, the analysis of plantar pressure data showed significantly higher arch index values in patients, which is associated with a flattened longitudinal arch [18].

Functionally, the medial longitudinal arch is another important shock-absorbing structure of the foot [26]. The overall shape of the kinematic arch height profiles shows a minimum arch height in mid stance and maximum height in late stance phase of walking, as described in previous studies [27–29]. It can be assumed that a PPV foot deformity with lowered medial longitudinal arch height loses elasticity and damping characteristics, which are necessary for a steady transfer of the body weight to the ground as well as for tolerating the resulting ground reaction forces [30]. Other authors [31, 32] describe a lowered medial longitudinal arch with a midfoot collapse. This is also characterized by a significantly lower peak hindfoot dorsiflexion and forefoot plantarflexion as well as greater forefoot supination. These characteristics were proven in JIA-PPV in static and walking condition by using the OFM. This result leads us to the assumption that the OFM calculation of the arch height does not provide secure results for our examination groups. It seems to be necessary to use an additional method and parameter such as the arch index of plantar foot pressure measurement.

### Impaired push-off phase

In JIA-PPV, the hindfoot is already in an everted, unlocked and flexible position at initial contact, and does not reach a rigid, inverted lever for propulsion in the late stance phases. Mosca [33] reported on the same characteristics in children with flexible flatfeet. In the push-off phases, consisting of terminal stance and pre-swing phase, JIA-PPV had a significantly lower peak hindfoot dorsiflexion, which was likely compensated by an enhanced forefoot dorsiflexion and a significantly lower peak forefoot plantarflexion. These findings are also described in previous studies in flatfeet population [9, 34, 13]. A further explanation for limited hindfoot motion in sagittal plane might be the lack of muscular stabilization of JIA-PPV: The m. tibialis anterior is hypertonic and the m. peroneus longus, m. tibialis posterior and m. triceps surae are hypotonic, which could lead to the distinctive forefoot dorsiflexion-position. In addition to considerable kinematic alterations, the kinetic data of the ankle showed a significantly reduced peak dorsiflexion moment and a lowered generated ankle power in push-off. The assumption of impaired push-off phases in JIA-PPV [4] is confirmed, but we could not expose a general lack of propulsion.

The results of this study show that a differentiated examination of the JIA foot is possible, as the OFM divides the foot into three segments. In comparison, the PIG models the foot as one rigid segment, which is the reason why Hartmann et al. [35] could only measure a significantly lower maximum plantarflexion of the ankle (foot/tibia) in JIA patients with polyarticular joint patterns. The OFM results showed that movement patterns between different segments are differently affected in JIA-PPV during upright standing and walking.

### Shift of forefoot/hindfoot movement in supination

As assumed before [4], the frontal forefoot angle profiles of patients appeared to be shifted in supination in both examination conditions. In peak angles, JIA-PPV showed an increased forefoot supination motion in loading response and a decreased peak pronation in terminal stance. In addition, the total pronation ROM was significantly enlarged in patients. In general, the frontal forefoot movement is important for shock absorption besides medial longitudinal arch and frontal hindfoot motion. The pronation and supination mainly occurs in the Chopart and Lisfranc joints [36] and influences the foot torsion during gait. The results of frontal and sagittal forefoot motion show that the foot torsion is restricted in JIA-PPV, but not absent. It is reported that deviations in hindfoot motion are often associated with misalignments of the forefoot [37]. In flatfoot, “rotationally opposite direction foot deformities“ occur [33]. Mosca [33] described this as a combination of deformities that include hindfoot valgus and forefoot supination deformity, which can be proved in this study. It can be assumed that the excessive hindfoot eversion provokes a restriction of forefoot pronation. Some similarities exist between results of this study and the work of Hoesl et al. [13]. They also suggested that the hindfoot eversion is accompanied by an excessive forefoot supination. In contrast, Twomey et al. [25] assumed that a decreased forefoot pronation is responsible for a lowered medial longitudinal arch rather than hindfoot eversion. In this study, we found both, a considerably increased hindfoot eversion motion and a reduced forefoot pronation in JIA-PPV.

Notably, the limited pronation and excessive hindfoot eversion of JIA-PPV may lead to a permanent mechanical stress, which might cause additional joint destructions in the long run. In addition, these misalignments could overburden muscles [13] or could provide future risks for other symptoms such as tibial stress syndrome [38] or anterior knee pain [39]. An early evaluation by 3d motion analysis and a resulting individual therapy for JIA-PPV could possibly prevent this. In tibialis posterior tendinopathy for example, a combination of stretching programs and strength training has been described as beneficial [40].

### Limitations

This study highlights the pathophysiology of JIA-PPV in static and walking condition using 3d gait analysis for the first time. However, there are some limitations in this study. First, the sample size of the patient group was small, because patients with JIA-PPV fulfilling all criteria of this study are rare. Secondly, the patients had at least one affected ankle joint, regardless of other inflamed joints. Thirdly, the evaluation of JIA-PPV was based on examination of the physicians with ultrasound imaging and tests by the physiotherapists. No X-rays were done for PPV diagnosis in order to avoid additional radiation. Finally, it cannot definitely be excluded that PPV derived from other factors than JIA.

## Conclusions

In conclusion, the results of 3d motion analysis of JIA induced pes planovalgus indicated a pathological hindfoot eversion motion during static and dynamic condition, which produce accessory symptoms such as a reduced arch height, increased forefoot supination and reduced propulsion effect of the ankle during stance phase of walking. We suggested that muscular imbalance and coordinative insufficiency caused by arthritis could be responsible for the increased hindfoot eversion from upright standing to stance phase of walking. So far, this foot deformity has only been described by clinical observations in relieving position or in upright standing under load using conventional methods such as clinical joint assessment with the neutral-zero method or static radiographs. However, 3d motion analysis allows a non-invasive, functionally meaningful monitoring and objective evaluation of dynamic function of JIA-PPV. The results of this study argue for an underestimated foot deformity through static examination methods and emphasize the dynamic evaluation of this foot deformity in clinical examinations. The outcomes recommend the early use of 3d motion analysis in JIA patients with affected ankle joints in order to detect malpositions and to treat them as early as possible. An adequate treatment concept could include for example physiotherapeutic treatment with the aim to relax hypertonic and strengthen hypotonic muscles and custom orthotic insoles. The documentation of the pathology in JIA-PPV and of the JIA disease course could be possible with the presented foot motion analysis. In clinical pratice, that functional diagnosis could lead to a targeted and enhanced, evidence-based medicine.

## Abbrevations

3d gait analysis: Three-dimensional gait analysis
AH: Arch height
AI: Arch index
CG: Control group
FF/HF: Forefoot to hindfoot motion
HF/TB: Hindfoot to tibia motion
JIA: Juvenlie idiopathic arthritis
JIA-PPV: Juvenile idiopathic arthrits induced pes planovalgus
Max: Maximum
Mdn: Median
Min: Minimum
OFM: Oxford Foot Model
PIG: Plug-in-Gait Model
PPV: Pes planovalgus
Q25: 25^th^ percentile
Q75: 75^th^ percentile
ROM: Range of motion
SD: Standard deviation
VAS: Visual Analog Scale
